# Protocol to develop a framework addressing barriers to utilization of elimination of mother- to -child transmission of HIV services among pregnant women and lactating mothers in Gauteng province

**DOI:** 10.1016/j.mex.2023.102351

**Published:** 2023-09-09

**Authors:** Ndivhuwo Mukomafhedzi, Takalani G. Tshitangano, Shonisani E. Tshivhase, Foluke C. Olaniyi

**Affiliations:** Department of Public Health, School of Health Sciences, University of Venda, Thohoyandou, South Africa

**Keywords:** Barriers, Elimination of mother-to-child transmission, Framework, Lactating mothers, HIV, Pregnant women, Utilization

## Abstract

Mother-to-child transmission of HIV remains the primary contributor to HIV infections in children, globally. Despite the progress made to reduce new HIV infections in children, barriers to utilization of the Elimination of Mother-to-Child Transmission service remain the bottleneck that affects the program's effectiveness. This study aims to develop a framework to address barriers to the utilization of the Elimination of Mother-to-Child Transmission of HIV services among pregnant women and lactating mothers in Gauteng province. A convergent parallel mixed methods design will be employed in phases. Phase 1(a) quantitative will be collected from pregnant women and lactating mothers to describe barriers associated with poor utilization of elimination of mother-to-child transmission services and a potential strategy to facilitate EMTCT utilization; phase 1(b) qualitative data will be collected to explore reasons for poor utilization of EMTCT services and perceived strategy to enhance women's utilization of elimination of mother-to-child transmission services. Quantitative data will be analyzed using Stata software version 14.0 and qualitative data will be analyzed thematically and then results will be integrated. Phase 2: will focus on the development of a framework; Phase 3: will validate the developed framework. The conclusion and recommendations will be based on the findings of the study.

## Graphical abstract

**Figure:**
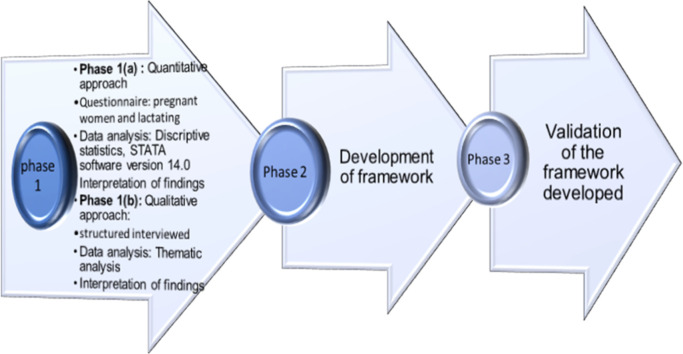

Specifications Table

**Table:** 

Subject area:	Medicine and Dentistry
More specific subject area:	*Maternal and Child Health*
Name of your protocol:	A framework addressing barriers to utilization of Elimination of Mother- to -Child Transmission of HIV services among pregnant women and lactating mothers in Gauteng Province
Reagents/tools:	*N/A*
Experimental design:	*N/A*
Trial registration:	*N/A*
Ethics:	*:* *Written informed consent will be sought from pregnant women and lactating mothers attending EMTCT services.*
Value of the Protocol:	• Describes in detail reproduceable methods to conduct systematic review of literature to identify existing strategies to improve the uptake of EMTCT. • Describes methods used to conduct qualitative phase of the study. • Describes methods used to conduct quantitative phase of the study. • Share reproduceable methods to develop a framework addressing barriers to the utilization of EMTCT services in Gauteng province and its validation.

## Description of protocol

### Introduction and background

Mother-to-child HIV transmission (MTCT) is a global healthcare issue, accounting for more than 90% of infections in children under the age of 15 [1]. In 2020, 38 million people were living with HIV worldwide, including 35.9 million adults and 1.7 million children under the age of 15. Although PMTCT Option *B*+ was introduced in 2013 to decrease MTCT, 150,000 new HIV infections in children aged 0–14 years were recorded in 2020 [2]. Preventable MTCT can be achieved with maternal ART usage throughout pregnancy and lactation, however, hurdles such as poor retention and adherence impede vertical transmission and compromise maternal and child health outcomes.

[3] indicates that the prevention of mother-to-child transmission (PMTCT) program in China has made significant strides in maternal HIV testing uptake and life-long ART for HIV-infected women and their children. However, early infant diagnosis (EID) remains a challenge, with MTCT rates decreasing from 7.4% to 3.6% between 2011 and 2020. This highlights the public health problem of MTCT of HIV in China, similar to other countries. India, Brazil, Cameroon, and Russia have successfully implemented PMTCT programs, but barriers like maternal retention and ART adherence remain a challenge [4,5]. Thirteen countries have been validated for achieving EMTCT of HIV, with Cuba being the first to be validated. Other countries include Thailand, Belarus, Moldova, Armenia, Anguilla, Montserrat, Cayman Islands, Bermuda, Antigua and Barbuda, and St Christopher and Nevis. Sri Lanka and the Maldives achieved EMTCT of HIV in 2019 [6,7].

Sub-Saharan Africa has a high HIV prevalence (67%), with women and girls accounting for 63% of new infections. This leads to high rates of HIV seroconversion among pregnant and postpartum women, making eliminating MTCT a challenge [8]. Barriers to MTCT persist at individual, community, and health-system levels [8]. Strategies should focus on low-to-middle-income countries, targeting vulnerable communities at three levels. Moreover, four African countries achieved over 90% PMTCT coverage in 2014, while 80% in South Africa and Rwanda [9].

In South Africa, the Department of Health introduced the PMTCT program in 2001, and the program began in 2002 with the supply of a single dosage of nevirapine (NVP) for both mother and newborn [10,11]. The World Health Organization reports that since introducing the PMTCT program, maternal, baby, and child mortality, as well as HIV prevention from mother to child, have improved [12]. HIV transmission from mother to child in South Africa has decreased from roughly 25–30% before 2001 to an estimated 1.4% in 2016. Furthermore, at the end of the 2018/19 financial year [13]. The rate had fallen to 0.74%.

MTCT of HIV is the main significant contributor to the high rate of HIV infections among children under the age of 15 years in Gauteng province of South Africa, estimated to be 51 800 in 2019. The Elimination of Mother-to-Child Transmission (EMTCT) program was introduced in South Africa as a vital initiative aimed at preventing the transmission of HIV from mothers to their babies during pregnancy, childbirth, or breastfeeding. This program offers a comprehensive package of services that include antenatal care, HIV testing and counseling, the provision of antiretroviral drugs to HIV-positive pregnant women, safe delivery practices, and postnatal care [12,13]. According to [14], all pregnant women should be tested for HIV during their first booking, and all HIV-positive women should be initiated into lifelong triple antiretroviral therapy. In addition, HIV testing is recommended at each routine Basic Antenatal Care (BANC) visit for all pregnant women who tested negative during ANC booking [14].

Moreover, all lactating mothers should receive an HIV test at delivery, 10 weeks post-delivery, and every three months while breastfeeding. According to South African PMTCT guidelines, Maternal Viral load (VL) monitoring for pregnant women and lactating mothers living with HIV at ANC visits or 3 months after ART initiation for all newly diagnosed with HIV, if viral load is below 50 copies, repeat VL at delivery, 6 months regardless of breastfeeding status, and every 6 months for all lactating mothers until the cessation of breastfeeding. This program has proved successful in reducing the number of babies born with HIV in Gauteng province from 8% in 2010 to less than 1% by 2016 [15]. However, poor utilization of these effective EMTCT services by women in the province remains a significant challenge. A study by [16] confirms that the uptake of EMTCT services remains low in the Ekurhuleni district, putting women and children at risk of deaths that could have been avoided. According to DHIS, only 66% of women attended ANC before 20 weeks gestation in quarter 2 2021/2022, which is below the target of 74% [17]. About 204 PCR-positive (Birth and 10 weeks PCR) results were reported in the Ekurhuleni district in 2022 [18]. Even though the number of PCR positives is low, mothers are still booking ANC after 20 weeks, which could put the health of the children at risk for seroconversion at a later stage. As a result, for the EMTCT program to be implemented effectively, strategies that encourage women to use ANC early are needed. Several factors were highlighted in the literature as barriers to the utilization of EMTCT services globally. Since contexts are different, it is essential to understand the barriers associated with EMTCT utilization in the City of Ekurhuleni to develop targeted interventions. Therefore, this study aims to determine the barriers to EMTCT services utilization among pregnant women and lactating mothers in Gauteng province of South Africa, in order to collect the baseline data upon which, the development of a framework to address the identified barriers will be based.

*The rationale of the study*South Africa has introduced several strategies to increase the utilization of EMTCT services and retention in care to eliminate MTCT since the implementing the PMTCT program in 2002 [19], [20], [21]. The introduced strategies include decentralization of healthcare services to improve access and utilization of quality healthcare services was one of the strategies, community-based interventions, integration of PMTCT services into routine antenatal care, task shifting and training, and involvement of male partners [13,22,23]. Community-based interventions such as home visits and peer support groups were found to be effective in increasing awareness about EMTCT services. The integration of PMTCT services into routine antenatal care improved accessibility and uptake among pregnant women. Task shifting and training enabled nurses to provide quality EMTCT services while increasing human resource capacity [22,24]. Additionally, in 2015 the Department of Health launched Gauteng Mentor Mothers Programme (GMMP) to combat health challenges and improve maternal and child health, increase the involvement of People Living with HIV and communities in the HIV response by providing HIV prevention education and supporting both HIV-negative and positive pregnant women and lactating mothers [25]. The National Department of Health further launched the postnatal club model (PNC) to improve postnatal care and postpartum HIV by integrating services [26,27]. This study aims to analyze the SWOT of each existing strategy outlined above, in order to develop a framework to Build on its strengths, overcome weaknesses, Explore opportunities, and Mitigate threats (BOEM) to address barriers to the utilization of EMTCT services in Gauteng province.

### Significance of the study

Effective use of EMTCT services through early antenatal booking and life-long ART initiation for those diagnosed HIV positive and an increase in lactating mothers' retention might all contribute to the global vision of an HIV-free generation. This study intends to describe the barriers to using EMTCT services that will give insight into the reasons behind late ANC booking and poor postpartum follow-up and explore potential interventions to improve EMTCT services utilization. This will not only contribute to the existing knowledge on EMTCT services, but it will also bring new knowledge specifically for the Ekurhuleni district. Once barriers to poor utilization of EMTCT services are identified, then the targeted framework to address barriers will be developed. Patients might benefit from the study as well, as they might be better informed about the advantages of EMTCT services. It might also encourage more patients to visit clinics since they would know they might obtain the services they needed for EMTCT.

As a result, the study results might assist the NDoH with evidence-based gathered barriers that impede the utilization of the EMTCT service in healthcare facilities. The study might provide valuable information that can be used to improve the utilization of EMTCT services. Government (policymakers) and healthcare professionals might also improve the service delivery at healthcare facilities that are not following the established processes for EMTCT services by applying the framework developed by this study or by addressing the shortcomings identified. The study might assist policymakers in developing public policies related to EMTCT services.

### Purpose of the study

The study aims to develop a framework to improve pregnant women and lactating mothers’ utilization of EMTCT services of HIV in Gauteng province, South Africa.

### Objectives of the study

The objectives of this study are described according to the phases in which the study will be conducted:

Phase 1 (a): Quantitative approach

• Describe barriers associated with poor utilization of EMTCT services among pregnant women and lactating mothers in Gauteng province.

Phase 1 (b): Qualitative approach

• To explore strategies to improve women's utilization of EMTCT services among pregnant women and lactating mothers.

Phase 1 (c): Meta-inferences and conceptualization of the findings

Phase 2: Framework development

• To develop a framework to enhance EMTCT utilization in Gauteng province.

Phase 3: Validation

• To validate the developed framework.

### Conceptual framework

This study will adopt the Knowledge, Attitude, and Practice (KAP) in phase 1(a) and Health Belief Model (HBM) in phase 1(b) to understand the barriers to the utilization of EMTCT services among pregnant women and lactating mothers. KAP is the leading theory behind the study but utilizes the construct of HBM to explore attitudes (the “A’) of KAP.

### Knowledge, attitude, and practice theory

A western scholar established the KAP theory in 1960 [28]. KAP is a health behavior change theory in which the changes in human behavior are divided into three processes, namely:

• The acquisition of knowledge
• The generation of attitudes and
• The formation of behavior

KAP presents the progressive relationship among knowledge, attitudes, and behavior as follows: knowledge is the foundation of behavior change, and belief and attitudes are the driving force of behavior change. Lack of knowledge of pregnant women and lactating mothers on the risks of MTCT, benefits of preventive interventions, such as prophylactic ARV drugs, and infant feeding options might impact EMTCT services utilization. Knowledge might be impacted by age, level of education, and occupation, which might also affect the utilization of EMTCT services. Lack of awareness of EMTCT services might influence pregnant women and lactating mothers’ decision to take action and utilize the services effectively. Pregnant women, lactating mothers, and healthcare providers’ attitudes and practices towards EMTCT services might also affect the utilization of EMTCT services. Applying the KAP theory can help us understand the reasons for the poor utilization of EMTCT services among women from the knowledge level, attitudes, and practice perspective.

Describing barriers associated with poor utilization of EMTCT services among pregnant women and lactating mothers will be studied using the knowledge, attitude, and practice (KAP)-theory [29]. HIV is a disease whose method of transmission is known and is mostly preventable, but its rapid spread is caused by a lack of knowledge and practices of HIV/AIDS and EMTCT in the general community [28].

Furthermore, [29] argue that the KAP theory can effectively enhance the patients' awareness of the disease, increase their rehabilitation consciousness, and promote them to adopt positive rehabilitation behavior, thus achieving the goal of changing the patients' KAP. Since HIV is a recurring condition with a protracted disease course, improvements in pregnant women and lactating mothers' education are essential during ANC first visit, follow-up visits, delivery, and post-delivery should be enhanced and sustained through knowledge, attitude, and practice (KAP).

The EMTCT strategy is about the elimination of mother-to-child transmission of HIV and at the same time meeting the nutritional requirements of the child, while also promoting safe sex. This requires prioritizing early prevention of HIV transmission through early maternal HIV screening and diagnosis, ARV initiation, and retention of EMTCT services post-delivery [14]. This implies that the effective implementation of EMTCT service is underpinned by KAP theory. Therefore, the attitudes part of KAP will be explored using the Health Belief Model construct of perceived susceptibility, perceived seriousness, perceived benefits, perceived barriers to behavior, perceived cues to action, and perceived self-efficacy [30].

• The Health Belief Model theory

**Fig. 1:**
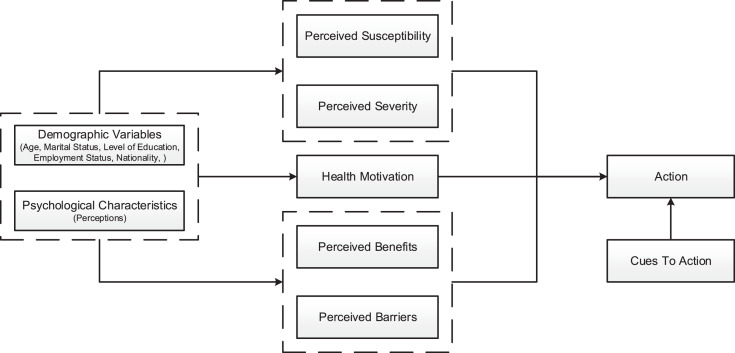
Health belief model diagram [34].

[31] states that the Health Belief Model (HBM) was established in the early 1950s by social scientists at the United States Public Health Service to explain why people fail to accept diseases, preventative measures, or screening tools for early disease diagnosis. According to the HBM, a person's belief in a personal threat of disease and confidence in the efficiency of the recommended health behavior or activity predicts the likelihood that such a person would adopt the practice. The model attempts to justify the premise that health-seeking behavior is influenced by the individual perceptions of threats posed by a health problem and the perceived benefits of taking action to minimize a health problem [32,33]. HBM is being applied in this study to identify/understand the reasons for poor utilization of EMTCT services among women by looking at their perception of susceptibility, seriousness, benefits, barriers to behavior, cues for action, and efficacy (Fig. 1). Barriers to EMTCT utilization negatively impact the program and play an important role in the women's behavior to take action to prevent MTCT.

The HBM in this study might assist in explaining why some pregnant women and lactating mothers take action to utilize MTCT services by attending early ANC, maternal HIV testing and re-testing of HIV throughout pregnancy and during the lactating period, adherence to ARV, while others utilize EMTCT services later or do not use it at all. The HBM might also assist in describing barriers associated with poor utilization of EMTCT services among pregnant women and lactating mothers. According to [30] the HBM is organized into three components that explain human behavior toward health. In this study, pregnant women, and lactating mothers' behavior towards utilization of EMTCT services include:

• Individual perceptions of pregnant women and lactating mothers concerning the utilization of EMTCT services.
• Modifying factors that could influence pregnant women and lactating mothers’ decision as to whether to utilize EMTCT services or not, namely.
• Demographic factors such as age, race, ethnicity.
• Socio-demographic factors such as social factors.
• Structural variables related to pregnant women and lactating mothers’ knowledge about the benefits of utilizing EMTCT services and the impact of poor utilization of EMTCT services.
• Variables affect pregnant women's likelihood and lactating mothers’ initiation of action to utilize EMTCT services.

HBM assumes that pregnant women and lactating mothers’ health beliefs are influenced by their perceptions of EMTCT services. However, modifying age, gender, marital status, parity, and educational status might influence their decision to utilize EMTCT services.

Variables such as perceived benefits might motivate pregnant women and lactating mothers to utilize EMTCT services. Perceived barriers such as the healthcare worker's negative attitude, privacy, and the lack of accessibility and availability of EMTCT services might influence the decision of not utilizing the services. The HBM is a conceptual framework that explores why people who are not ill might take specific actions to prevent illness while some do not take measures. The HBM might motivate pregnant women and lactating mothers to take action through EMTCT services utilization during pregnancy and post-delivery to reduce the MTCT of HIV in their children.

## Definition of concepts

### Barriers

[35] define a barrier as contributing factor or a variable that prevents an individual from using the services or using them properly. In this study, a barrier refers to personal, environmental, social, and political technological factors limiting access to women's utilization of EMTCT services.

### Elimination of mother-to-child transmission of HIV (EMTCT)

According to this study, elimination of mother-to-child transmission refers to having zero transmission of HIV from mother-to-child HIV during pregnancy, delivery, and lactating period.

### EMTCT service utilization

Utilization refers to the point of entry into the service. In this study, utilization of EMTCT services refers to pregnant women accessing and receiving ANC services, HIV testing, ART initiation and lactating mothers attending EMTCT follow-up services, HIV testing, receiving infant feeding information, early infant diagnosis through PCR testing for all exposed children routinely from birth to up to 18 months.

### Framework

[36] defines a framework as a particular set of rules, ideas, or beliefs that you use to deal with problems or decide what to do. In this study, the framework will include ideas or beliefs from pregnant mothers, healthcare workers, literature & experts to address barriers to EMTCT services utilization.

### Lactating mothers

The medical dictionary defines lactation as the formation of milk in the breasts during the period following childbirth. A mother is defined as a woman who bears a child [36]. In this study, a lactating mother is a woman breastfeeding a child following childbirth.

### Pregnant women

A pregnant woman is defined as a woman who carries a developing foetus in her uterus from conception to birth or a woman who carries a developing embryo in her womb for nine months [37]. In this study, a pregnant woman refers to a woman carrying a developing foetus attending EMTCT service before the delivery of the child.

## Methodology

The study will employ a convergent parallel mixed method design.

### Research approach and design

A mixed-method research design involves collecting and integrating quantitative and qualitative data [38]. Phase 1(a) of this study will employ quantitative research approach, descriptive design; phase 1 (b) will employ qualitative research approach, explorative design approach. In this study, both quantitative and qualitative data will be collected simultaneously. The researcher adopted a mixed-method technique to increase the validity of the findings and to get generalizability of the results as well as the depth that will arise from the results. As each method aims to address a different study purpose, the mixed-methods approach will also give complementarity to the outcomes.

### Outline of the study process

The study will be conducted in three phases. The first phase will begin with data collection using questionnaires and interviews simultaneously with pregnant women and lactating mothers Phase 1(a) the study will employ a quantitative descriptive design to describe barriers associated with poor utilization of elimination of mother-to-child transmission services among pregnant women and lactating mothers and a potential strategy to facilitate EMTCT utilization; phase 1(b) qualitative data will be collected to explore reasons for poor utilization of EMTCT services and perceived strategy to enhance women's utilization of elimination of mother-to-child transmission services. Data will be then analyzed separately, and the results will be merged. The second phase will involve the development of a framework. The third phase will then validate the developed framework.

## PHASE 1: emperical situational analysis study

***PHASE 1 (a) – Quantitative Research approach and design***

This section of the study will focus on quantitative study. Then outline the study setting, population, sampling, measurement instrument, data collection plan, data analysis and validity and reliability.

### Study setting

**Fig. 2:**
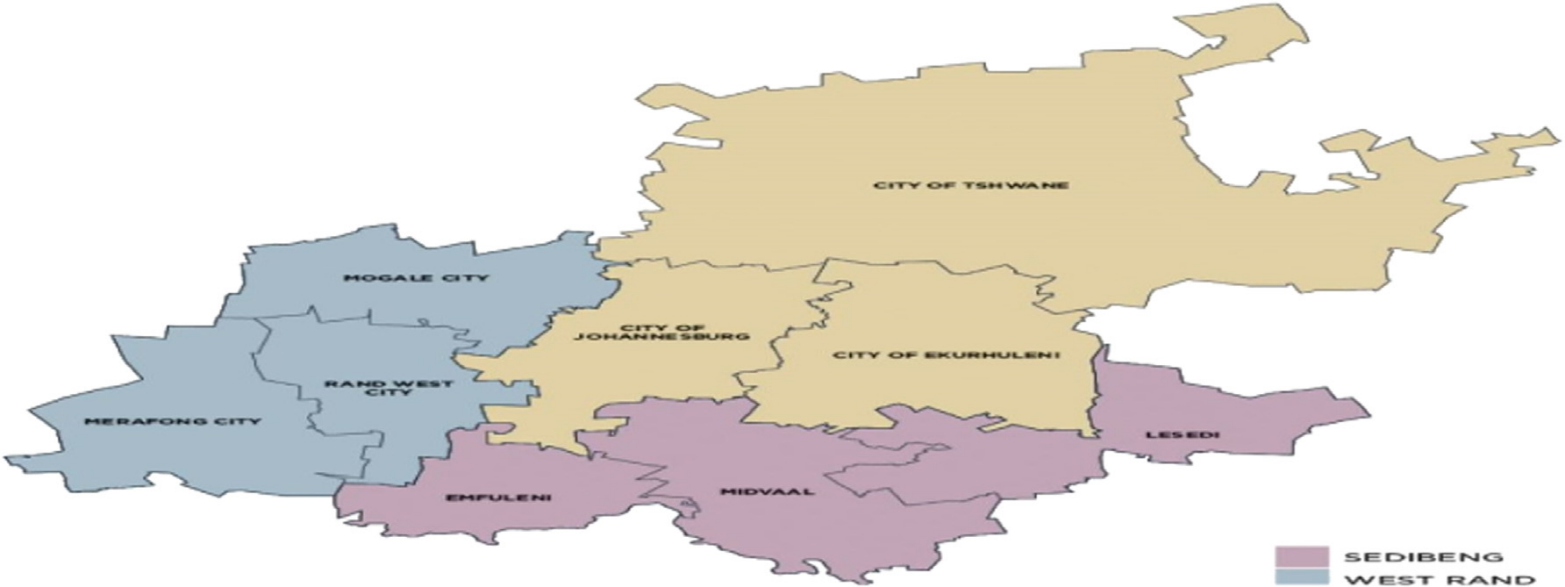
Gauteng province map [40].

The study will be conducted in The City of Ekurhuleni, Gauteng province, South Africa. Gauteng province is one of the nine provinces in South Africa with three metropolitan municipalities: the City of Tshwane, the City of Johannesburg, and the City of Ekurhuleni, and two districts, West Rand and Sedibeng district (Fig. 2). The City of Ekurhuleni is the second largest district in Gauteng province in the west to the east part, with 3 Sub-District serviced by 93 fixed clinics and six (6) local hospitals. Gauteng province has the highest influx of migrants standing at approximately 1048 440 reported in a period of 2016–2021 [39]. Moreover, the province is viewed as the economic hub of South Africa and attracts refugees, asylum seekers, economic migrants, and domestic migrants from rural areas such as Limpopo, Kwa-Zulu Natal, and Eastern Cape.

The City of Ekurhuleni is considered one of the most densely populated areas in the province, and the country with the estimated population of 3 858 147 [15]. It accounts for nearly a quarter of Gauteng's economy and the network of roads is found within the municipality; for instance, airports, rail lines, telephones, electricity grids, and telecommunications.

### Study population

A research population refers to the total number of precisely defined cases, records, events, units, collections of items, corporation units, or individuals who possess specific characteristics and can be included as research subjects [38,41]. All pregnant women attending ANC services and lactating mothers attending EMTCT follow-up visits within the proposed study period at the six facilities in the City of Ekurhuleni will form the study population.

The catchment population served by the selected facilities is estimated at 1 050 670 consisting predominantly of black people from different ethnic groups (Northern Sotho, Tsonga, Venda, Zulu, Malawians, Mozambicans, and Zimbabweans). The total number of pregnant women tested for HIV is 54 594, and 7.8% (4 280) tested positive for HIV [42]. The study population will include all pregnant women attending ANC and lactating mothers attending EMTCT services in the selected facilities

### Sampling

**Sampling will be done in multi-stages by sampling the municipality, clinics, pregnant women and lactating mothers.**

### A sampling of the metropolitan municipality

One metropolitan municipality will be purposively sampled for this study. The city of Ekurhuleni municipality was chosen as the area of study for these reasons: similar studies have been conducted in other municipalities in the past [16,43], and there is low uptake of EMTCT services resulting in high positivity rate of children below ten years; lastly, there is a poor retention in lactating mothers postpartum which leads to a missed diagnosis of exposed children. Moreover, Ekurhuleni municipality is densely populated and has high cases of children living with HIV [17]. The researcher will sample the City of Ekurhuleni municipality since Wits Reproductive Health Institute (WRHI) has offices within the district that render Health systems strengthening, which supports EMTCT services.

### Sampling of clinics

In this study, six clinics will be purposively sampled. The researcher has generated a list of clinics that provide EMTCT services. The study will be conducted at six clinics (three CHC, three PHC) in Ekurhuleni North, Ekurhuleni East, and Ekurhuleni South (Winnie Mandela, Esangweni CHC, Nokuthela Ngwenya CHC, Daveyton Main clinic, Phola Park CHC, and Kempton Park civic center clinic). Three clinics are situated in the north sub-district, one in an urban area and two in a semi-rural area. Two clinics are situated in the east subdistrict and the south subdistrict. These clinics were selected because of their location and number of EMTCT services attendance ranging from the highest, middle and the lowest; in urban, semi-urban and rural areas. Moreover, all clinics within the district have the same procedure for EMTCT services; therefore, data from those facilities can be generalized around the Ekurhuleni district.

### Sampling of pregnant women and lactating mothers

For this study, non-probability sampling will be explicitly used as convenience sampling. Convenience sampling allows researchers to choose respondents with the desired/required characteristics based on accessibility and availability [44].

### Sample size

The average monthly ANC attendance for first visit is 2 250 pregnant women; the average monthly attendance of lactating mothers is 2 236 in the City of Ekurhuleni. A sample size of 339 (pregnant women) and 339 lactating mothers will be targeted. The sample size is calculated using Slovin's formula (Table 1). N is the total number of pregnant women attending ANC visit and lactating mothers in the selected facilities, n is the sample size and e is the accepted error level. The accepted level of error, e, is 0.05. The estimated overall sample size was 338 of all pregnant women and 339 of lactating mothers will be drawn from an estimated total population of 2 240 (pregnant women) and 2 236 (lactating mothers), with a 95% confidence interval (refer to the table below for the calculation).

**Table 1:** Pregnant women and lactating mothers estimated population size.

Pregnant women	Lactating mothers
*n* = N / (1+Ne^2^) = _2240______ 1+ (2240 × 0.05^2^) = __2240___________ 1+ (2240 × 0.0025) = _2240_ 1 + 5.625 = 2240 6.625 =338	*n* = N / (1+Ne^2^) = _2236______ 1+ (2236 × 0.05^2^) = __2236___________ 1+ (2236 × 0.0025) = _2236_ 1 + 5.59 = 2236 6.59 =339

### Inclusion criteria

All pregnant women and lactating mothers aged 18 years and above attending EMTCT services (ANC visits, postnatal follow-up care, and EPI services) who are willing to participate will be included in the study.

### Exclusion criteria

During data collection, all pregnant women and lactating mothers attending the clinic for other services (those attending integrated management of childhood illness (IMCI) and acute illness services) will be excluded from the study.

### Measurement instrument

A self-administered structured questionnaire will be used. The questionnaire will be developed to focus on barriers to the utilization of EMTCT services among pregnant women and lactating mothers. The developed questionnaire was adopted from the study's findings conducted by [16]. It will be developed in English and translated into two local languages, namely: Isi-Zulu, and Northern Sotho, and each participant will be given their language preference. The data collection tool will be translated using language experts because not all respondents understand English. The questionnaire will comprise six sections. The sections in the questionnaire will be dictated by the conceptual framework, namely, KAP and HBM conceptual framework. Section 'A' will consist of demographic and socioeconomic information, section 'B' will consist of Knowledge about EMTCT services, section 'C' will consist of practices regarding EMTCT services, section 'D' attitudes, and beliefs towards EMTCT services, section 'E' barriers to EMTCT services utilization and section 'F' potential strategies to facilitate utilization of EMTCT services.

### Pre-test

The researcher will pre-test the questionnaire before the data collection process to ascertain whether it is free from error and able to collect accurate and relevant data. [38] describe pretesting as a process of objective evaluation of the questionnaire and provisional analysis before conducting the main research. The questionnaire will be pretested using participants who meet the inclusion criteria. Participants will be recruited from one clinic in Ekurhuleni North 1. The pre-test will be conducted to determine whether the proposed questionnaire will successfully obtain the relevant information while assessing whether the items in the questionnaire are straightforward and consistent or need to be phrased [38].

### Validity

Validity means that the data collected to address the research questions is a close representation of the aspects of the social reality of the study in question [41]. The validity of the measuring instrument will be tested by assessing face, content, and construct validity.

### Face validity

[45] indicated that face validity verifies that the instrument appears valid and gives the appearance of measuring what it is meant to measure. In this study, the questionnaire will be given to the promoter and two co-promoters who are experts in the field of study to examine the questionnaire to ensure that the instrument's content is aligned with the study objectives. The questionnaire will then be modified where necessary based on the feedback from the promoters and pre-testing.

### Content validity

Content validity assesses how well the instrument represents all the major components of the variables to be measured. Therefore, those who are experts (assistant director of Maternal and child health, maternal and child health district coordinator, medical doctor, and midwives offering EMTCT services and stakeholders) in the field will be allowed to assess the questionnaire for content validity to improve the content of the data collection tool.

### Reliability

Reliability is a measure of quality and consistency to estimate the reliability of a given test [46]. This study will ensure the instrument's reliability by conducting a pre-testing using 10% of the total sample size. Once the pre-testing is conducted, questionnaires will be modified and re-administered to the same population within two weeks. Therefore, Cronbach's alpha coefficients will be calculated to measure the measuring instrument's internal reliability and evaluate its internal consistency (where coefficients equal to or greater than 0.70 will be regarded as reliable).

### Bias

Selection bias will be minimized by recruiting all pregnant and lactating mothers for the study. Response bias will be addressed by collecting data off-duty so that the participants do not perceive the researcher as a healthcare provider. A questionnaire will be translated to IsiZulu and Northern Sotho to minimize response bias. Analytical bias will be minimized by ensuring that more than one statistical test is used, and all collected data will be included during data analysis.

### Plan for data collection and recruitment process

Once approval has been granted by the University of Venda Research Ethics Committee, letters requesting permission to conduct a study will be written to the Gauteng Department of Health and the City of Ekurhuleni municipality. There will be proper arrangements with Operational Managers in the clinics before data collection, and a private room will be prepared as a data collection room. The researcher will collect data with the assistance of two research assistants. Data will be collected in the healthcare facilities selected for the study. The researcher will recruit all pregnant women and lactating mothers attending ANC and postnatal visits enrolled in EMTCT services. Participants will be briefed about the study in a private room and informed that their participation is voluntary and that they can withdraw at any time without consequences. Due to Covid-19 restrictions, participants will be briefed in a group of 10 participants per session. Those willing to participate will be asked to sign the consent form, which will explain the purpose of the study, including the benefits and risks. All participants who signed the informed consent form will be issued questionnaires. After the completion, the researcher will collect all the questionnaires from the participants and ensure all questions are answered before the participant leaves the room.

### Plan for data analysis

Before data analysis, the researcher will check all the questionnaires for completeness, consistency, and plausible values. All questionnaires will be kept in a safe place where they will be inaccessible to unauthorised individuals. Data will be cleaned, coded, and entered into an Excel spreadsheet. After entering data into an Excel spreadsheet, it will be imported into Stata software version 14.0 for statistical analysis. Demographic variables and attitude scores will be summarized using descriptive summary measures, i.e., means (standard deviation) or median (minimum-maximum), calculated to describe the sample. Data will be checked for normality distribution and outliers for continuous numerical variables.

Descriptive statistics will be used to analyze data. Chi-Square, which is known as a statistical test used to compare observed data and can only be used on actual numbers that are in the form of proportions, percentages, frequencies, and means, will be used [38]. The p-value of the chi-square test will determine the statistical significance of the relationship between the variables [38,47]. The p-value of <0.05 will indicate a statistically significant relationship between two dependable variables and the outcome. Analyzed data will be presented as proportions, graphs, and tables. Data analysis will be categorized into KAP constructs, where ‘A’ will utilize the HBM constructs.

PHASE 1 (b) – Qualitative study

This section will focus on the qualitative part of the study. This section will be assessed to solicit/explore perceived solutions to barriers associated with poor utilization of EMTCT services. Then outline the methodology from approach, design, setting, population, sampling, plan for data collection, and data analysis and trustworthiness.

### Study setting

The study will be conducted in Gauteng province in South Africa. The City of Ekurhuleni municipality will be selected for this study. The study will be conducted at the six clinics (three CHC, three PHC) situated in Ekurhuleni North (Winnie Mandela clinic, Esangweni CHC and Kempton Park civic center), Ekurhuleni East (Daveyton Main Clinic and Nokuthela Ngwenya CHC), Ekurhuleni South (Phola Park CHC).

### Study population

The study population will be consisting of pregnant women and lactating mothers utilizing EMTCT services from the selected clinics within the proposed District.

### Sampling

Pregnant women and lactating mothers utilizing EMTCT services will be targeted to participate in the study.

### Sampling of pregnant women and lactating mothers

The researcher will target pregnant women and lactating mothers utilizing EMTCT services within the proposed study period. A convenience and purposive sampling strategy is employed for the study to ensure equal representation [19]. Pregnant women and lactating mothers utilizing EMTCT services will be recruited to the study, encouraging voluntary participation. A total of 30 pregnant women and lactating mothers will be targeted during data collection. A total of 12 pregnant and 18 lactating mothers will interviewed from the six selected. However, the actual number of pregnant women and lactating mothers who will be interviewed will be determined by data saturation.

### Data collection tool

A semi-structured interview guide will be used as a data collection tool (See Appendix B). An interview guide is developed in English. The interview guide will consist of two central open ended question exploring barriers to utilization of EMTCT services and possible strategy to enhance utilization of EMTCT services among pregnant and lactating mothers. Further questions will be probed from the responses given by the central question. An open-ended question refers to an inquiry in which potential respondents are not given options for choosing their answers [21]. Instead, it is informative and allows respondents to express their views spontaneously.

### Trustworthiness of data

In qualitative research, trustworthiness ensures the accuracy of the findings. The researcher will strictly use the following to maintain the study's trustworthiness: credibility, dependability, Confirmability, transferability, and authenticity.

### Credibility

This process is essential for this qualitative research, and the researcher will provide an in-depth description of the complexities of variables and interactions rooted in data obtained from the setting.

### Dependability

The researcher will ensure the process is logical, well-documented, and audited. The researcher will use the coding process, including open, axial, and selective coding [48]. The first spiral stage, open coding, created the early dependability conditions. After that, the researcher will apply selective coding, which entails selecting one major category that connects all the codes from the analysis and captures the essence of the research. Themes and sub-themes will then be drawn up during selective coding.

### Confirmability

The researcher will ensure that data support the findings, conclusions, and recommendations and an agreement between the interpretation and the actual evidence [45]. Therefore, the researcher will achieve this through the supervisors, who periodically act as an oversight and auditing body throughout the study. In addition, the data analysis process adopted by this study will maintain objectivity.

### Transferability

External validity will help the researcher ask whether the findings can be transferred to another setting or group [47]. Although [49] purports that traditionalists see the generalization of qualitative studies as a weakness because of different populations and settings, this study claims to be the same.

### Authenticity

Refers to how the researcher can express the participants' feelings, experiences, and emotions [47]. Therefore, the researcher will quote participants' experiences when writing narratives during data analysis.

### Plan for data collection

A plan for data collection provides clear guidance on how data will be collected, and the kind of data collected. There will be proper arrangements with Operational Managers in the clinics before data collection, and a private room will be prepared as a data collection room. The researcher will recruit all pregnant women and lactating mothers attending ANC and postnatal visits enrolled in EMTCT services. The researcher will collect data with the assistance of 2 research assistants. The interviews will be conducted in a private room in the selected facilities proposed for the study. Data will be collected using English because the participants targeted understand English.

### Preparation for data collection

The researcher will recruit all pregnant women and lactating mothers attending ANC and postnatal visits enrolled in EMTCT services in the selected facilities proposed for the study to participate. Participants will be given information about the study in a private room and allowed to decide if they want to participate. Participants will be informed that participation in the study is voluntary, and those willing to participate will be requested to consent before the interview. Participants will be reassured that their personal information will only be shared with those directly involved in the study. Participants will be informed that data may be used for a similar study if there is a similar study. Participants will be requested to record the interview sessions using an audiotape. If any of the participants refused, his/her interview should not be tape-recorded. The researcher will also use field notes to supplement language-focused data. An interview session will be face-to-face with participants taking approximately 30–45 min. Data will be collected for 4 to 6 weeks, determined by saturation.

### Plan for data analysis

According to [38], data analysis involves meaningfully categorizing, arranging, and summarizing data.

Qualitative data will be analyzed following the steps by [45].

Step 1 *managing and organizing data*

Once data is collected, Audio files will be transcribed verbatim and translated into English. Data will be organized in a form that can be analyzed.

Step 2 *Coding*

The researcher will find a pattern and produce an explanation using both inductive and deductive reasoning [45]. Transcripts will be reviewed by the researcher and coded using thematic analysis. The analysis will involve multiple readings of transcripts by the researcher to identify the themes. Emerged themes will be previewed for reliability. Transcripts will be re-coded if a new theme emerges or if a theme is redefined.

Step 3 *Explanatory codes and categorizing*

The researcher will try to understand the meaning of data, and coding might change as the researcher gains a deeper understanding of the phenomenon.

Step 4 *Data analysis and interpretation of the results*

Interpretation of the results will be based on the researcher's understanding of the phenomenon. Data will be analyzed using NVivo version 11, a qualitative data analysis (QDA) computer software package. The KAP/HBM construct-based strategies to mitigate the barriers identified in Phase 1 will be confirmed or strengthened during the exploration process of the existing frameworks.

Phase 1 (c): **Meta-inferences and conceptualization of the findings**

Data analysis from quantitative and qualitative data will be done separately. Therefore, quantitative, and qualitative data interpretations will be connected using meta-inference. The overall conclusions from qualitative and quantitative studies will be categorized into KAP/HBM constructs in this phase. These overall conclusions will be presented in tabular form.

### PHASE 2 - Framework development methodology

In this phase, a framework to improve EMTCT utilization by pregnant and lactating mothers in Gauteng province will be developed using SWOT, a systematic literature review, and the BOEM model.

### SWOT analysis

In this study phase, the KAP/HBM constructs conclusions derived from Phase 1(c) will be further analyzed using the Strength, Weaknesses, Opportunities, and Threats (SWOT) analysis. The findings of the SWOT analyses will be used as the basis for developing the KAP/HBM construct-based framework to ensure that the proposed model addresses the pitfalls of the existing frameworks [50]. The Systematic review of the literature and BOEM model will be used to develop the KAP/HBM construct-based framework.

### A systematic review of the literature

The systematic review entails selecting relevant studies, extracting the necessary data into the form developed to summarize the included studies, assessing the biases of each study, determining the quality of the available evidence, and constructing tables and text that synthesize the evidence [51]. Systematic reviews are a crucial element of evidence-based healthcare [52], and their strength is underpinned by transparency and replicability [53,54]. The objective of the systematic literature review will be to explore the landscape of methodological frameworks and barriers to the utilization of EMTCT services, where PRISMA-ScR (Preferred Reporting Items for Systematic Reviews and Meta-Analyses Extension for Scoping Reviews) standards and guidelines will be followed. As a result, understanding and interpreting the scientific evidence will necessitate comprehending the available levels of evidence, with systematic reviews of literature about the barriers to utilizing EMTCT services.

A systematic review search will be performed using various databases, e.g., PubMed, EBSCOhost, science direct, and Google Scholar. The literature search heading will be EMTCT utilization. The researcher will identify and select similar studies. To ensure that the final data extraction is from high-quality and trustworthy studies, the researcher will include or exclude the study depending on the similarity of the studies. Studies that are different from the study will be excluded. Data will be synthesized and might be or not combined from different studies.

### BOEM model

The BOEM model is best known for its ability to assist in developing a framework that can be used to achieve the overall goals by building on the Strengths, overcoming Weaknesses, exploring Opportunities, and minimizing Threats [55]. Thus, this BOEM model will be adopted after the SWOT analysis has identified the Strength, Weaknesses, opportunities, and Threats from qualitative and quantitative findings. In this study, the KAP/HBM construct-based framework to be developed will focus on overcoming threats and weaknesses to improve the utilization of EMTCT services among pregnant women and lactating mothers. In addition, the framework to be developed will aim to minimize the chances of failing to achieve the main goal: to address barriers to using EMTCT services among pregnant women and lactating mothers.

**PHASE 3 - VALIDATION AND REFINEMENT OF THE DEVELOPED FRAMEWORK**

In this phase, the researcher aims to validate the developed KAP/HBM construct-based framework to improve the utilization of EMTCT services. This section will cover validating the developed framework using the Delphi technique.

### Study design

The Delphi technique will validate and refine the developed framework to improve EMTCT utilization by pregnant and lactating mothers. Thus, the researcher will invite the experts to review the proposed framework for validation [56]. The Delphi technique has proven to be a reliable measurement instrument in developing new concepts, and it seeks the opinion of a group of experts to address the gaps.

### Study setting

The study has a variety of geographical areas as it allowed local and international experts to give inputs to refine the proposed framework.

### Targeted population

Experts in the HIV/EMTCT include the assistant director of Maternal and child health, the maternal and child health district coordinator, medical doctors, and midwives offering EMTCT services, and stakeholders. The researcher will identify the discipline, number, and content of groups and establish the method and procedures of communication.

### Sampling

A non-probability sampling method will be used, specifically expert sampling. The expert sampling method will be used because the researcher seeks consent from experts in the field of the study.

### Sample size

Twelve to twenty experts from across the globe in health will be expected to participate in the study.

### Inclusion criteria

The study will include all experts who agree to participate in the study.

### Data collection tool

The Likert scale form for rating the proposed framework will be used as a data collection tool. A 7-point Likert scale (1: strongly, 2: disagree, 3: somewhat disagree, 4: undecided, 5: somewhat agree, 6: agree, 7: strongly) agree will be used to collect data.

### Plan for data collection

An email with the topic of the study summary, including the findings from phases one and two, the purpose of the study, inclusion criteria, and sample size, will be sent to invite the expert to participate in the study. The researcher and promoter's details will be included in the email for those wanting to participate in the study. Participants who might show interest in the study will be requested to reply to the email with a "yes.” After that, the researcher will send the appropriate paperwork. Participants will be requested to validate the developed framework within 14 days. Data collection will be divided into rounds one and round two rounds. However, if the consensus is not reached in round two, round three will be included. Any information provided by experts as part of this study will remain confidential. After completing the study's findings, the participating experts will send feedback on the study findings.

### Round one

Experts will rate the framework, and the comments received will be analyzed and used to refine the framework.

### Round two

Round two will be conducted following the results from round one. Data collected in round two will be analyzed.

### Plan for data analysis

Data will be entered into an Excel spreadsheet and coded, and if there are any missing data and mistakes, the database will be corrected. After entering data into an Excel spreadsheet, it will be imported into Stata software version 14.0 for statistical analysis. Demographic variables and attitude scores will be summarised using descriptive summary measures, i.e., means (standard deviation) or median (minimum-maximum), calculated to describe the sample.

### Ethical consideration

The following ethical considerations will be considered in this study:

### Ethical clearance

The study proposal and tools were submitted to the Department of Public Health and the School of Public Health for quality assessment. After that, they were submitted to the University of Venda Human Research Ethics Committee for ethical clearance [FSH/22/P.H./17/902].

Permission to conduct the study.

Permission was sought in writing from the Gauteng Provincial Department of Health and relevant health facilities managers. Letters will be submitted to the City of Ekurhuleni district Department of Health to inform them that the researcher has been granted permission to conduct the study in the district by the Provincial Department of Health.

### Informed consent

To ensure that all participants voluntarily consent to participate in the study, they will be provided with a participant information letter explaining the study's aim. It will also be clearly explained that they have the right not to participate in the research and should not in any way feel threatened by not participating in the research. If they decide to participate in the study, they will be asked to sign the informed consent form. Each participant will be allowed to refuse to participate. Hence all participants should ideally be willing participants. Participants in the in-depth interviews will also be allowed to consent to have the interviews recorded. The researcher will explain to the participants whether they can choose to have their interview recorded.

### Confidentiality

To ensure confidentiality, the researcher will use participant code to label data collected instead of names, whereas an alias will be used to identify participants during the interview. Information provided during the study will only be made available to persons involved. The researcher will be sensitive to issues such as confidentiality, and participants will be informed that the results of this study will also be published in journals and conference proceedings, both locally and internationally. However, no personally identifiable information will be published in any publication.

### Anonymity

The actual names of the participants will not be used in the study. Each participant will be given a number, for example, "1″ The numbers will be used when discussing the study's findings. Furthermore, any information that can lead to the identification of participants will not be used in this study [44].

### Principle of non-maleficence

The participants' emotional, psychological, and physical well-being will be protected throughout the study. Sensitive words and questions which might negatively affect the well-being of the participants will be avoided when interacting with the participants. Social distancing will be maintained to protect participants from Covid-19 during the study, and all covid-19 protocols will be adhered to at all times.

### Plan for dissemination and implementation of results

The study results will be published in peer-reviewed, accredited international and national journals. A copy of the thesis will be made available at the University Library. Other copies will be shared with the Gauteng Provincial Department of Health and other Provinces. The study's empirical results will be presented at national and international conferences and research seminars.

## CRediT authorship contribution statement

**Ndivhuwo Mukomafhedzi:** Conceptualization, Methodology, Validation, Data curation, Writing – original draft, Visualization, Investigation. **Takalani G. Tshitangano:** Supervision, Validation, Writing – review & editing. **Shonisani E. Tshivhase:** Supervision, Validation, Writing – review & editing. **Foluke C. Olaniyi:** Supervision, Validation, Writing – review & editing.

## Declaration of Competing Interest

The authors declare that they have no known competing financial interests or personal relationships that could have appeared to influence the work reported in this paper.

## Data Availability

No data was used for the research described in the article.

## Appendix C Supplementary materials

Image, application 1

Image, application 1

Image, application 1

Image, application 1

Image, application 1
