# Supplementary material for: Protocol to develop a framework addressing barriers to utilization of elimination of mother- to -child transmission of HIV services among pregnant women and lactating mothers in Gauteng province
Source: MethodsX. 2023 Sep 9;11:102351. doi: 10.1016/j.mex.2023.102351 (PMC10565866; doi:10.1016/j.mex.2023.102351)
Supplement: Supplementary file 1 [file mmc1.docx]

**APPENDICES**

**Appendix A: Questionnaire**

**Topic: A framework addressing barriers to utilization of Elimination of Mother- to -Child Transmission of HIV services among pregnant women and lactating mothers in Gauteng Province**

**SECTION A**

**DEMOGRAPHICAL INFORMATION**

Please mark your selection with a click in the appropriate box.

1 Age ……...

2 Level of education

| No formal education |  | 1 |
| --- | --- | --- |
| Senior certificate (Grade 12/Matric) |  | 2 |
| Higher Certificate/Diploma/Bachelor's degree |  | 3 |
| Postgraduate diploma/degree |  | 4 |
| Other, please specify: |  | 5 |

3 Population Group

| Asian |  | 1 |
| --- | --- | --- |
| Black |  | 2 |
| Colored |  | 3 |
| Indian |  | 4 |
| White |  | 5 |
| Not willing to say |  | 6 |

4 Nationality

| South African – Birth |  | 1 |
| --- | --- | --- |
| Non-South African |  | 2 |

5 Marital Status

| Married |  | 1 |
| --- | --- | --- |
| Single |  | 2 |
| Divorced |  | 3 |
| Widow |  | 4 |
| Other, please specify: |  | 5 |

6 Who do you live with

| Spouse |  | 1 |
| --- | --- | --- |
| Parents |  | 2 |
| Friends |  | 3 |
| Other, please specify: |  | 4 |

7 Religion

| Christianity |  | 1 |
| --- | --- | --- |
| Islamic |  | 2 |
| African spirituality |  | 3 |
| Other, please specify: |  | 4 |

8. Employment status

| Self-employed |  | 1 |
| --- | --- | --- |
| Employed |  | 2 |
| Unemployed |  | 3 |
| Other, please specify: |  | 4 |

9. Distance traveled between the clinic and your home

| 1-25kn |  | 1 |
| --- | --- | --- |
| 26-50km |  | 2 |
| More than 50km |  | 3 |

10. What type of transportation do you use to come to the clinic

| Public transport |  | 1 |
| --- | --- | --- |
| Private car/own |  | 2 |
| Walking |  | 3 |

11. How long does it take you to travel to the clinic?

| Less than 1 hour |  | 1 |
| --- | --- | --- |
| 1-2 hours |  | 2 |
| >2hours |  | 3 |

12. Obstetric information

| Parity – Number of children |  |
| --- | --- |
| Gravida – Number of Pregnancy |  |
| Age of the youngest child <1 yr. |  |

13. What was your pregnancy’s gestation (age) when you made your first ANC visit?

| Less than 20 weeks | Yes | No |
| --- | --- | --- |
| More than 20 Weeks | Yes | No |

**Experience information**

2.1 How was your most recent experience when you came for EMTCT services?

| Good experience |  | 1 |
| --- | --- | --- |
| Neutral |  | 2 |
| Bad experience |  | 3 |

2.2 How was the attitude of the healthcare professionals towards you?

| Positive attitude |  | 1 |
| --- | --- | --- |
| Neutral |  | 2 |
| Negative attitude |  | 3 |

**Section B**

**KNOWLEDGE ABOUT EMTCT**

1. Are you aware of EMTCT services

| Yes | 1 |
| --- | --- |
| No | 2 |

2. What is your source of information

| Health Care providers | 1 |
| --- | --- |
| Relatives and friends suffering from HIV | 2 |
| Media (TV,radio,health magazine, pamphlets | 3 |

3. Early participation in EMTCT ensures the well-being of the mother and baby

| Yes | 1 |
| --- | --- |
| No | 2 |

4. EMTCT increases understanding of viral load suppression, identification of danger signs, and act promptly

| Yes | 1 |
| --- | --- |
| No | 2 |

5. EMTCT provides a learning opportunity to enhance adherence to ART during and after pregnancy.

| Yes | 1 |
| --- | --- |
| No | 2 |

6. EMTCT provides an opportunity to learn about STI and HIV prevention (PrEP, PEP)

| Yes | 1 |
| --- | --- |
| No | 2 |

**SECTION C**

**PRACTICES REGARDING EMTCT SERVICES**

1. Where do you prefer to attend EMTCT services from?

| Hospital | 1 |
| --- | --- |
| Mobile clinic/health center | 2 |
| Private doctor | 3 |

2. On average, how many EMTCT visits do you miss during pregnancy and postnatal?

| None | **1** |
| --- | --- |
| 1-2 | **2** |
| 3-5 | **3** |
| More than 5 | **4** |

3**.** Challenges faced when utilizing EMTCT services?

| Cultural issues (pregnancy disclosure) | **1** |
| --- | --- |
| In adequate knowledge about benefits of EMTCT services | **2** |
| Negative staff attitude | **3** |
| Unknown pregnancy | **4** |
| Unclear clinic processes | **5** |

4.Do you suffer from other chronic illness?

| Yes | **1** |
| --- | --- |
| No | **2** |

5**..** If yes, Which ones

| Hypertension | **1** |
| --- | --- |
| Diabetes | **2** |
| Arthritis | **3** |
| Others | **4** |

**SECTION D**

**ATTITUDE AND BELIEFS TOWARDS EMTCT SERVICES**

|  | Statement | **Agree** | **Disagree** |
| --- | --- | --- | --- |
| 1 | There is no benefit in utilizing EMTCT services | 1 | 2 |
| 2 | I feel frustrated about having HIV infection | 1 | 2 |
| 3 | Regular EMTCT follow-up is important | 1 | 2 |
| 4 | I believe that EMTCT services is for women with first time pregnancy | 1 | 2 |
| 5 | I feel comfortable when nurses provide individualised health education about EMTCT | 1 | 2 |

**section E**

**ENVIRONMENTAL DETERMINANTS CONTRIBUTING TO LOSS TO FOLLOW UP**

**Barriers to EMATCT Service Utilization. Which of the following have been significant barriers for you in attending your follow-up for EMTCT services in the past year?**

1. Other medical or physical condition?

| Yes | 1 |
| --- | --- |
| No | 2 |

2. Financial barriers

| Transportation cost | **1** |
| --- | --- |
| Unemployment | **2** |

| Yes | 1 |
| --- | --- |
| No | 2 |

3. Unfriendly hospital staff /Staff attitude

4. Long waiting time at the clinic/hospital

| Yes | **1** |
| --- | --- |
| No | **2** |

5. Nurse/Counsellor shortages

| Yes | 1 |
| --- | --- |
| No | 2 |

6. I was unhappy with the care I received here in the past

| Yes | **1** |
| --- | --- |
| No | **2** |

7**.** Lack of partner support

| Yes | **1** |
| --- | --- |
| No | **2** |

8. Shortage of treatment

| Yes | **1** |
| --- | --- |
| No | **2** |

9. Do you ever forget to come for an EMTCT follow-up?

| Yes | **1** |
| --- | --- |
| No | **2** |

| Yes | **1** |
| --- | --- |
| No | **2** |

10. Have **y**ou ever missed an EMTCT appointment?

11. Long distance to the health facility

| Yes | **1** |
| --- | --- |
| No | **2** |

**SECTION F. POTENTIAL STRATEGIES TO FACILITATE EMTCT UTILIZATION**

Which of the following would help you to attend your regular follow-up?

1. Pre-appointment reminder a week before your scheduled date of visit (by phone, email, or text message)

| Yes | 1 |
| --- | --- |
| No | 2 |

2. Having a mobile clinic offering EMTCT services that come to your area of residence once every month on a specific day

| Yes | 1 |
| --- | --- |
| No | 2 |

| Yes | 1 |
| --- | --- |
| No | 2 |

3. More education on the importance of follow up

4.Having more education on EMTCT services

| Yes | 1 |
| --- | --- |
| No | 2 |

5.Having transportation services that brings you to the clinic and takes you back home

| Yes | 1 |
| --- | --- |
| No | 2 |

6.Networking with other women attending EMTCT services for peer support group

| Yes | 1 |
| --- | --- |
| No | 2 |

**7.** Male partner involvement.

| Yes | 1 |
| --- | --- |
| No | 2 |

**THANK YOU FOR YOUR PARTICIPATION!**
