# Supplementary material for: Protocol to develop a framework addressing barriers to utilization of elimination of mother- to -child transmission of HIV services among pregnant women and lactating mothers in Gauteng province
Source: MethodsX. 2023 Sep 9;11:102351. doi: 10.1016/j.mex.2023.102351 (PMC10565866; doi:10.1016/j.mex.2023.102351)
Supplement: Supplementary file 2 [file mmc2.docx]

**Appendix B: Interview guide**

1. In your own opinion, what might be the reasons for you not to utilize EMTCT services?
2. What do you think should be done to improve your EMTCT services utilization?
