# Supplementary material for: Protocol to develop a framework addressing barriers to utilization of elimination of mother- to -child transmission of HIV services among pregnant women and lactating mothers in Gauteng province
Source: MethodsX. 2023 Sep 9;11:102351. doi: 10.1016/j.mex.2023.102351 (PMC10565866; doi:10.1016/j.mex.2023.102351)
Supplement: Supplementary file 3 [file mmc3.docx]

**Appendix C: Informed consent**

**Title of the Research Study**: **A framework addressing barriers to utilisation of Elimination of Mother- to -Child Transmission of HIV services among pregnant women and lactating mothers in Gauteng Province**

**Principal Investigator/s/ researcher**: (*Mukomafhedzi Ndivhuwo, Doctoral Degree in Public Health*)

**Co-Investigator/s/supervisor/s** : (*Prof TG Tshitangano, Dr SE Tshivhase, Dr FC Olaniyi*)

**Brief Introduction and Purpose of the Study**: To develop a framework to address barriers to utilization of Elimination of Mother to Child Transmission of HIV (EMTCT) services among pregnant women and lactating mothers. Mother-to-child transmission (MTCT) of HIV is currently a global healthcare problem. According to the United Nations (2021), MTCT of HIV is responsible for more than 90% of HIV infections in children under 15 years. Therefore, effective use of EMTCT services through early antenatal booking and life-long ART initiation for those diagnosed HIV positive, as well as an increase in lactating mothers' retention and early infant diagnoses (EID), might all contribute to the global vision of an HIV-free generation. This study intends to describe the barriers to using EMTCT services and explore potential interventions to improve the EMTCT services.

**Outline of the Procedures**: A self-administered questionnaire will be used to collect data from HIV positive and HIV negative pregnant women, lactating mothers attending EMTCT follow-up. Closed-ended questions will be included in the survey. A sample size of 339 pregnant women and 338 lactating mothers will be targeted during data collection from six selected health facilities in Ekurhuleni municipality. The data collection tool for patients will consist of for sections that will be dictated by the theoretical framework, namely, KAP and HBM conceptual framework. The second phase of research will target healthcare providers offering EMTCT services within the proposed study period. An in-depth interview will be used to collect data from healthcare providers offering EMTCT services at the selected facilities.

**Risks or Discomforts to the Participant**: There is no harm expected of the participants during their participation in the study.

**Benefits**: Publications will be done in accredited journals nationally and internationally. Knowledge gained from this study might provide valuable information for a community or the general population, and the healthcare system might be improved.

**Reason/s why the Participant May Be Withdrawn from the Study**: Participants may withdraw from the study, and there will be no adverse consequences should they choose to do so.

**Remuneration**: There will be no remuneration for participating in the study.

**Costs of the Study**: No costs will be covered by the participants.

**Confidentiality**: The researcher will maintain confidentiality by ensuring that the information provided by the participants in this study is not shared with other people who are not part of the research project

**Research-related Injury:** No anticipated injuries:

Persons to Contact in the Event of Any Problems or Queries:

Please contact the researcher (081 527 6091/073 056 0656), my promoter or the University Research Ethics Committee Secretariat on 015 962 9058. Complaints can be reported to the Director: Research and Innovation, Prof GE Ekosse, on 015 962 8313 or Georges

Ivo.Ekosse@univen.ac.za

**CONSENT**

Statement of Agreement to Participate in the Research Study:

- I have been informed by the researcher (Mukomafhedzi N) about the nature, conduct, benefits, and risks of this study - Research Ethics Clearance Number:
- I have also received, read, and understood the above-written information (*Participant Letter of Information*) regarding the study.
- I am aware that the study results, including personal details regarding my sex, age, date of birth, initials, and diagnosis, will be anonymously processed into a report.
- In view of the research requirements, I agree that the data collected during this study can be processed in a computerized system by the researcher.
- At any stage, I may withdraw my consent and participation in the study without prejudice.
- I had sufficient opportunity to ask questions and (of my own free will) declare myself prepared to participate in the study.
- I understand that significant new findings developed during this research related to my participation will also be made available to me.

Full Name of Participant Date Time Signature

**I, ……………………. …………… ………… …………………..**

(N Mukomafhedzi) herewith confirm that the above participant has been fully informed about the nature, conduct and risks of the above study.

Full Name of Researcher

**…………………………….**  Date**……………….**  Signature**…………………**

Full Name of Witness (If applicable)

**…………………………**  Date **……………….**  Signature**…………………..**

Full Name of Legal Guardian (If applicable)

**………………………..** Date**………………**  Signature**…………………**
