# Supplementary material for: Protocol to develop a framework addressing barriers to utilization of elimination of mother- to -child transmission of HIV services among pregnant women and lactating mothers in Gauteng province
Source: MethodsX. 2023 Sep 9;11:102351. doi: 10.1016/j.mex.2023.102351 (PMC10565866; doi:10.1016/j.mex.2023.102351)
Supplement: Supplementary file 4 [file mmc4.docx]

**Appendix D: Request for permission from the Gauteng Provincial Department of Health**

University of Venda

Private Bag X5050

Thohoyandou

0955

Department of Health

P/Bag X085

Marshalltown

2107

Dear Sir/Madam

RE: REQUEST FOR PERMISSION TO CONDUCT RESEARCH

I am seeking permission to conduct Doctoral research (Doctor of Philosophy in Public Health) at healthcare facilities in the City of Ekurhuleni Municipality.

I am a Doctoral student at the University of Venda who intends to conduct a study at Health facilities in partial fulfillment of my degree (Doctor of Public Health).

I wish to conduct my study in the City of Ekurhuleni, where pregnant women and lactating mothers, who are utilizing EMTCT services in the selected clinics will be interviewed. Development of a framework to address barriers to the utilization of Elimination Mother to Child Transmission of HIV services among pregnant women and lactating mothers in Gauteng Province.

A self-administered questionnaire will be used for data collection with patients, and face-to-face interviews will be used for data collection with healthcare providers offering EMTCT services the information gathered will be treated with confidentiality. A summary of the report will be made available to the Department of Health after the study.

Your positive response will be highly appreciated.

You can contact me on:

Contact: 081 527 6091/073 056 0656

Email: mndivhuwo@webmail.co.za

Yours faithfully

N Mukomafhedzi
